# Virtual histological assessment of the prenatal life history and age at death of the Upper Paleolithic fetus from Ostuni (Italy)

**DOI:** 10.1038/s41598-017-09773-2

**Published:** 2017-08-25

**Authors:** Alessia Nava, Alfredo Coppa, Donato Coppola, Lucia Mancini, Diego Dreossi, Franco Zanini, Federico Bernardini, Claudio Tuniz, Luca Bondioli

**Affiliations:** 1 Dipartimento di Biologia Ambientale, Università di Roma “La Sapienza”, Rome, Italy; 2 Servizio di Bioarcheologia, Museo delle Civiltà, Rome, Italy; 3 Università degli Studi di Bari “Aldo Moro”, Bari, Italy; 4 Museo di Civiltà Preclassiche della Murgia Meridionale, Ostuni, Italy; 5 Elettra - Sincrotrone Trieste S.C.p.A., Basovizza, Trieste, Italy; 6 Centro Fermi, Museo Storico della Fisica e Centro di Studi e Ricerche “Enrico Fermi”, Piazza del Viminale 1, 00184 Roma, Italy; 7 Multidisciplinary Laboratory, The “Abdus Salam” International Centre for Theoretical Physics, Strada Costiera 11, 34014 Trieste, Italy; 8 Centre for Archaeological Science, University of Wollongong, Northfields Ave, Wollongong, NSW 2522 Australia

## Abstract

The fetal remains from the Ostuni 1 burial (Italy, ca 27 ka) represent a unique opportunity to explore the prenatal biological parameters, and to reconstruct the possible patho-biography, of a fetus (and its mother) in an Upper Paleolithic context. Phase-contrast synchrotron X-ray microtomography imaging of two deciduous tooth crowns and microfocus CT measurements of the right hemimandible of the Ostuni 1b fetus were performed at the SYRMEP beamline and at the TomoLab station of the Elettra - Sincrotrone laboratory (Trieste, Italy) in order to refine age at death and to report the enamel developmental history and dental tissue volumes for this fetal individual. The virtual histology allowed to estimate the age at death of the fetus at 31–33 gestational weeks. Three severe physiological stress episodes were also identified in the prenatal enamel. These stress episodes occurred during the last two months and half of pregnancy and may relate to the death of both individuals. Compared with modern prenatal standards, Os1b’s skeletal development was advanced. This cautions against the use of modern skeletal and dental references for archaeological finds and emphasizes the need for more studies on prenatal archaeological skeletal samples.

## Introduction

Hominin dental development can offer important insights into hominin evolutionary trajectories^1–10^. Studies of fetal and perinatal individuals can shed light on the life history - in its broadest sense, the history of biological events in someone’s life - of both the mother and the child during gestation. However, studies focusing on prenatal dental development are rare in the palaeoanthropological literature.

Fetal or neonatal remains of fossil hominins are rarely found. Even the anatomically modern human fossil record includes a relatively scant number of fetal or neonatal specimens^11^. Neonates have been collected from Cro-Magnon^12^ (France, ca 27 ka), Kostenki^13^ (Russia, 23–29 ka), Krems-Wachtberg^14^ (Austria, 26–27 ka), Neuwied-Irlich^15^ (Germany, ca 12 ka), Qafzeh^16^ (Israel, 90–120 ka), Wilczyce^17^ (Poland, ca 13 ka). Nazlet Khater^18^ (Upper Egypt, ca 37 ka), Ostuni 1b^19, 20^ (Os1b, Apulia, Italy, ca 27 ka), and possibly Nataruk^21^ (Turkana Lake, Kenya, ca 10.5–9.5 ka) represent the only known Upper Paleolithic fetuses. All three of these fetuses were found inside their mother’s pelves.

**Figure 1:**
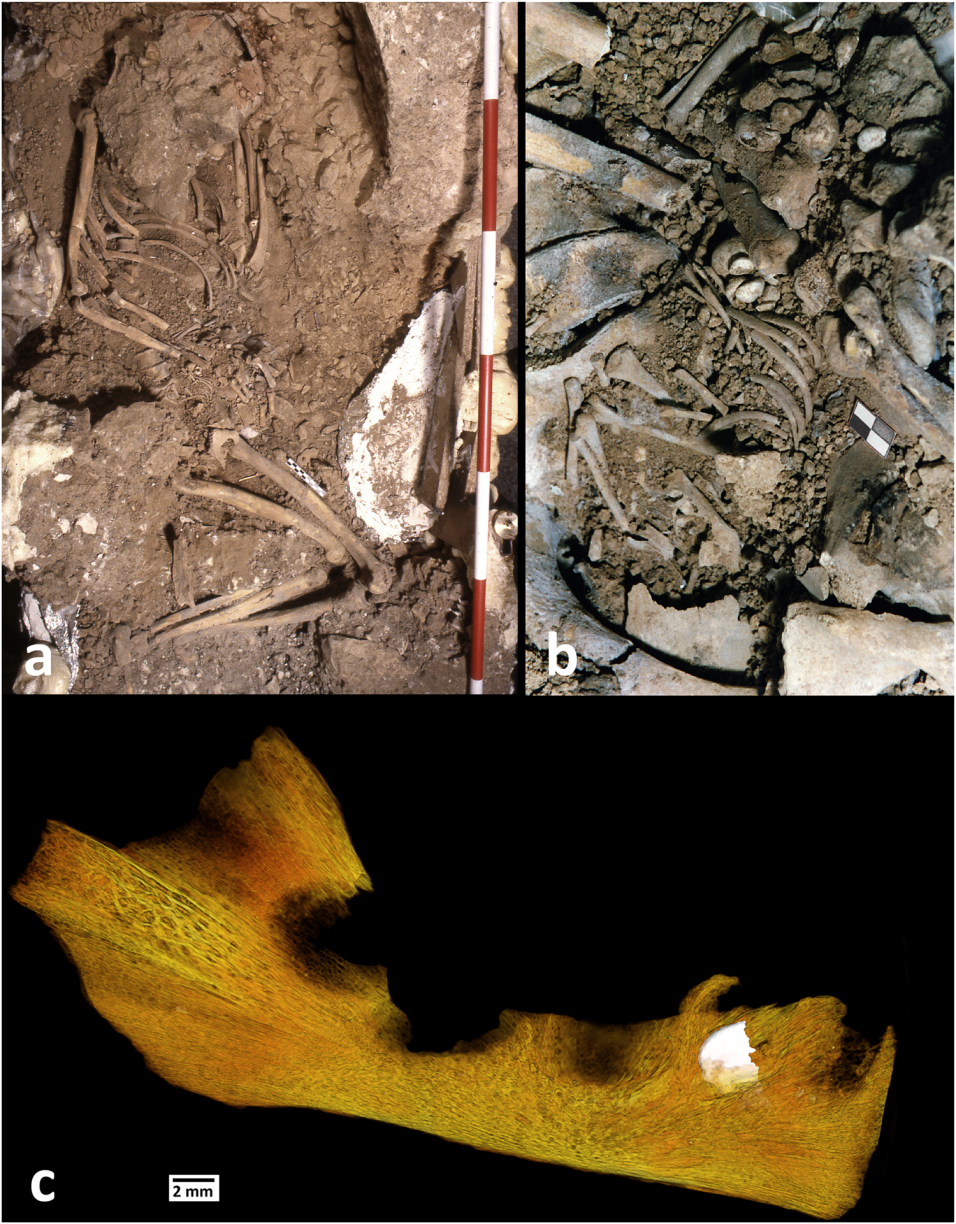
The Ostuni 1 burial in the Santa Maria di Agnano cave: (**a**) the burial during excavation (photograph by E.Vacca); (**b**) an enlargement of the pelvic region of Os1 with the fetus Os1b taken during excavation (photograph by E.Vacca); (**c**) Virtual volume rendering of the right hemimandible of Os1b in lateral view. The lower right lateral deciduous incisor (LRi2) is visible through the bone transparency.

The Upper Paleolithic funerary complex at Ostuni was discovered in 1991 in the Santa Maria di Agnano cave (Ostuni, Apulia, Italy). To date, two primary burials, Ostuni 1 and Ostuni 2, have been discovered at the site. Ostuni 1 grave contained the skeleton of a young woman (Os1). She was 20 years of age or younger and in the advanced stages of pregnancy at time of death^22, 23^. Her skeleton was discovered in an excellent state of preservation, and was richly adorned with hundreds of perforated shells around her wrists and covering her head. The shells covering her head were pasted together with red ochre^22^. She was buried lying on her left side, in a crouched position, with her right forearm obliquely placed on her abdomen (Fig. 1a). Os1 showed no macroscopic signs of stress or trauma, aside from slight periodontitis and a small amount of dental calculus on the anterior lower dentition^23^. Os1 individual has been dated to 27,810-27,430 cal BP^20^. An almost complete fetus (Os1b) was found inside the pelvic region of Os1 (Fig. 1b). Os1b was also found in an excellent state of preservation. The remains included the partially formed crowns of six *in situ* or isolated anterior deciduous incisors^23^. The placement of the fetus was consistent with the position typically seen during pregnancy. Os1b’s head was located inside the small pelvis while all of its postcranial remains were found, articulated, inside the great pelvis^23^. The Ostuni 2 grave contained the skeleton (Os2) of an unsexed adult, in a crouched position. Os2 was poorly preserved. This paper will focus on the dental remains of Os1b, with the express purpose to report the enamel developmental history and dental tissue volumes for this individual as well as to refine its age at death.

Previous study of Os1b was carried out by Vacca and coworkers^23^, based on gross skeletal and dental analyses compared with age at death standards from modern collections^24^ (Supplementary Table S1). A significant amount of variability (between 28 and 38 fetal weeks) in developmental stages was observed across the different anatomical regions, with the most frequent estimation being between 30–38 weeks of age. The cranial bones seem to be in an earlier developmental stage than the appendicular skeleton^23^. Furthermore, the analysis of the relative limb proportions could be suggestive of an earlier developmental stage, thus highlighting some discordance with modern skeletal series. Vacca and coworkers’ conclusions suggest a final age at death estimate of 34 to 36 gestational weeks, with a high likelihood of true age being closer to 36 gestational weeks^23^.

Vacca and coworkers’ morphological study of Osb1^23^ did not include dental histological analyses due to the destructive nature of dental sectioning. Access to high resolution synchrotron light microtomography now allows for non-destructive histological analysis of the mineralized tissues (virtual histology)^8, 10, 25–27^. This approach provides direct estimate of the enamel chronologies, allowing for accurate assessment of age at death in individual still growing at the moment of death^1, 4, 28^. This technique of incremental ageing, which relies on individual physiological rhythmicity, has the advantage to overcome the use of skeletal indirect methods, that are necessarily based on reference populations’ growth standards.

Here we report the virtual histomorphometry and the virtual volume rendering of three of the anterior deciduous crowns of Os1b: the upper left deciduous central incisor (ULi1), the lower right deciduous central incisor (LRi1) and the lower right deciduous lateral incisor (LRi2). The lower right deciduous lateral incisor is still embedded inside the body of the mandible (Fig. 1c). The aim of this study is to enrich our knowledge of the prenatal biology and the patho-biography of this fetus (and consequently of its mother as well) and to refine its age at death estimate.

## Results

**Figure 2:**
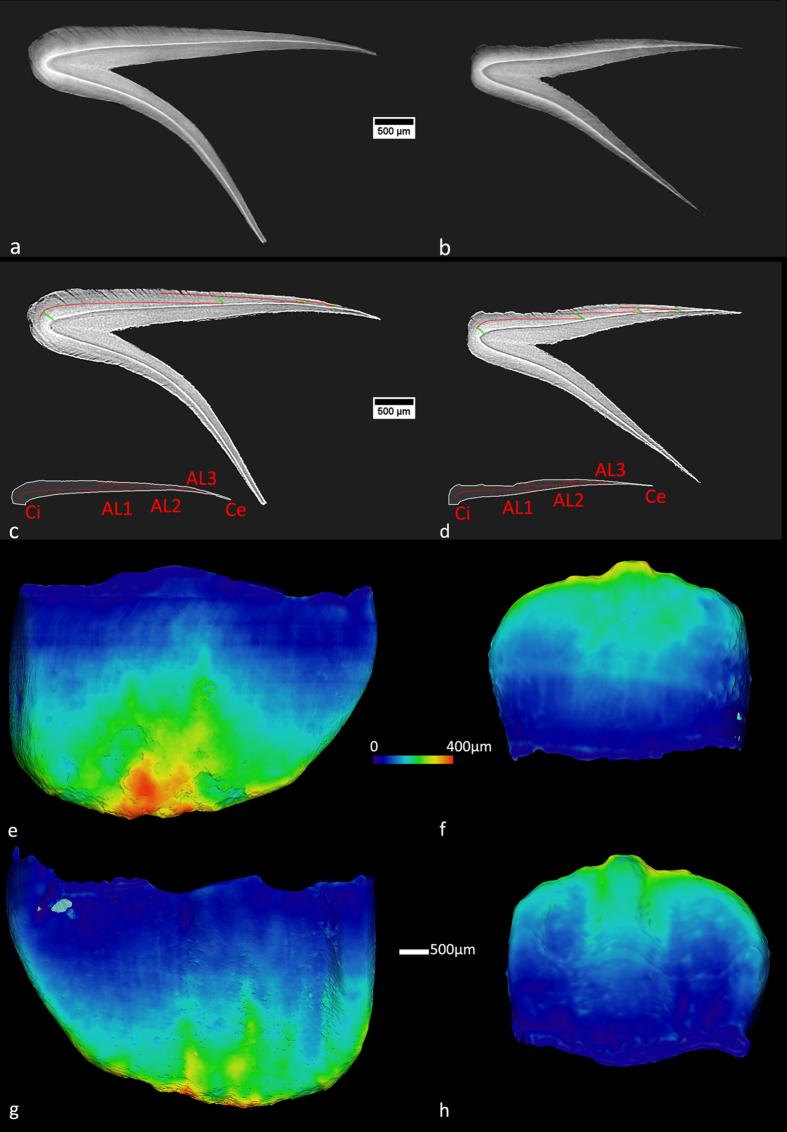
Virtual histology and volume rendering of Os1b’s ULi1 and LRi1: (**a**,**b**) virtual histological sections of ULi1 (**a**) and LRi1 (**b**) crowns;(**c**,**d**) digitally enhanced sections of the ULi1 (**c**) and LRi1 (**d**) crowns. The ALs are highlighted in red and the prism paths in green; (**e-h**) virtual 3-D reconstruction of the two deciduous central incisors. Enamel thickness topographical variation is rendered by a chromatic scale from dark blue to red. ULi1 labial (**e**) and lingual (**g**) views; LRi1 labial (**f**) and lingual (**h**) views.

Two virtual histological sections passing through the bucco-lingual plane at the tip of the dentine horn of the ULi1 and LRi1 have been selected from the synchrotron X-ray microtomography (SRμCT) measurements and are shown in Fig. 2a,b. The enamel prisms are discernible as faint structures, and their orientation is well defined in different portions of the crowns. In the enamel of both central deciduous incisors, three clear Accentuated Lines (ALs marked as AL 1, AL 2 and AL 3; Fig. 2c,d), representing episodes of severe physiological stress^29, 30^, have been detected on the buccal side of the teeth. Some of these ALs are also present, but less visible, on the lingual side of the teeth.

Table 1 reports the measurements along the enamel prisms from the Enamel Dentine Junction (EDJ) to the ALs, the estimated time represented by each distance in days and weeks, and the total Crown Formation Time (CFT). These estimates were created using the regression formula developed by Nava and coworkers^31^. The time interval between AL 1 and AL 3 is identical and sums to 46 days in both crowns. The relative chronology of AL 2 differs by only 1 day between the two teeth. The Enamel Extension Rate (EER, i.e. the rate of differentiation of secretory ameloblasts, or the speed at which ameloblasts on the secretory front are recruited along the EDJ^32^) has been calculated by dividing the lengths of the four segments along the EDJ by the corresponding number of days. The EER is also reported in Table 1. The EER of both teeth decelerates towards the cervix except in the last portion of enamel. This evidence contradicts the expectation of a decrease of EER values in the last forming enamel^32^. It is possible that a portion of the last secreted enamel has been lost post-deposition due to its poor mineralization. To estimate how much enamel is missing in both crowns, the EERs of the third segments were used (see Supplementary Methods).

**Table 1:** Measurements in μm along the prisms from the EDJ to the ALs and transformation in days and weeks estimated applying the Nava and coworkers^31^ regression formula. The 95% confidence intervals limits are shown. See text and Supplementary Methods online for the estimate of the adjusted crown formation time. Ci = Crown initiation, Ce = Crown end, EER = Enamel Extension Rate. *Values of EER used to correct the final age at death, see the text and Supplementary Methods online for detail.

Tooth	Landmarks	Prism Length (μm)	Length on the EDJ (μm)	Prediction from regression (days)	EER	95% upper limit	95% lower limit	Prediction from regression (weeks)	95% upper limit	95% lower limit
Li1R	Ci - AL 1	109.3	1313.9	24	56.2	26	23	3.4	3.7	3.2
	AL 1 - AL 2	130.9	721.5	28	25.8	31	27	4.0	4.5	3.9
	AL 2 - AL 3	84.4	500.8	18	27.7*	20	18	2.6	2.9	2.5
	AL 3 - Ce	90.1	762.7	19	40.1	21	19	2.8	3.1	2.7
	CFT			89		98	87	12.7	14.0	12.4
	Adjusted CFT			98		107	96	14.0	15.3	13.7
Ui1L	Ci - AL 1	156.3	2201.7	34	65.8	37	33	4.8	5.3	4.6
	AL 1 - AL 2	132.1	1031.9	29	36.5	31	27	4.1	4.5	3.9
	AL 2 - AL 3	79.0	386.9	17	22.9*	19	16	2.4	2.7	2.3
	AL 3 - Ce	49.7	590.3	11	53.7	12	10	1.5	1.7	1.5
	CFT			91		99	86	13.0	14.1	12.3
	Adjusted CFT			108		116	103	15.4	16.6	14.7

**Figure 3:**
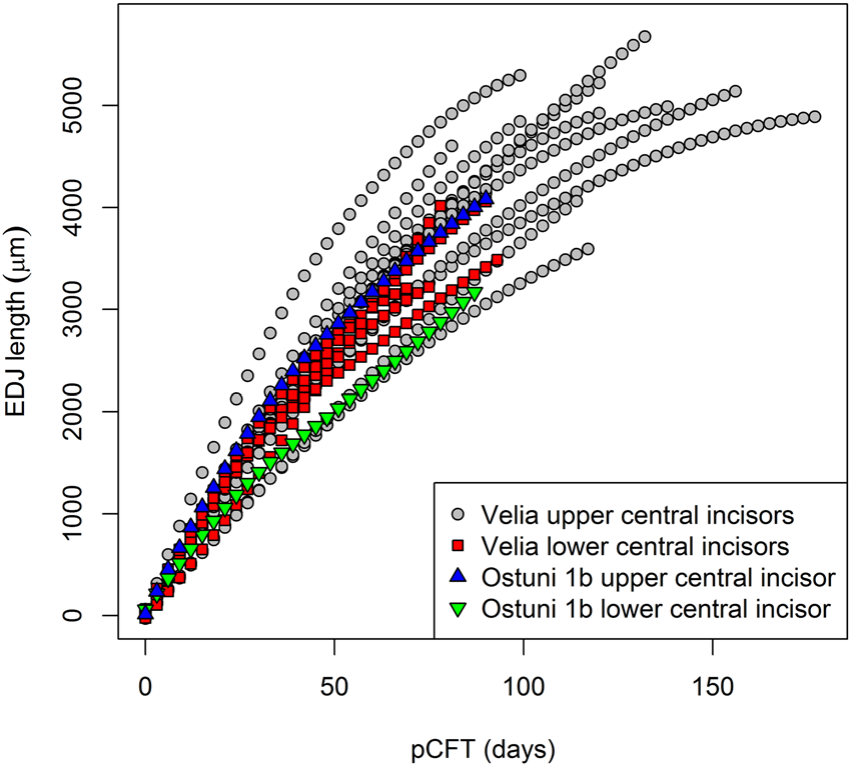
CFT variation with reference to the EDJ length of the Os1b mandibular (green triangles) and maxillary (blue triangles) deciduous central incisors. Same data reported by Nava *et al*.^31^ for the Roman imperial series of Velia (grey circles and red squares). Each profile was calculated with a locally weighted polynomial regression fit. See methods section for details.

The CFT variation with reference to the EDJ length of the Os1b mandibular and maxillary deciduous central incisors can be seen in Fig. 3, together with the CFTs and EDJ lengths reported by Nava *et al*.^31^ for the Roman imperial series of Velia^33^ (Campania, Italy), which includes a number of preterm individuals. The CFTs and EDJ lengths profiles of Os1b fall within the range of variation observed for Velia and follow the same trajectories.

The crown tissue volumes and the EDJ surface areas of Os1b’s deciduous teeth are presented in Table 2. Here they are compared with the fully developed deciduous dental crowns of extant human^34, 35^ (EH) and with Lagar Velho^35^ (early Upper Paleolithic) and La Madeleine^34^ (late Upper Paleolithic) specimens. Enamel thickness topographic variation is shown in Fig. 2e–h. The chromatic scale, increasing from dark blue to red, shows that the upper incisor has thicker cuspal enamel in reference to LRi1. The distribution of the enamel thickness is partially altered by minor erosions on the enamel surface.

**Table 2:** Crown tissues volumes and EDJ surface of the Os1b teeth compared to extant human (EH) values and to the Lagar Velho (Early Upper Paleolithic) and La Madeleine (Late Upper Paleolithic); the percentages represent the relative amount of dental tissues of the Os1b in reference to the comparative available values.

		ULi1	%	LRi1	%	LRi2	%
^1^Ve	Ostuni 1b	6.63		3.16		2.23	
	Lagar Velho^+^	28.78	23.0%	—	—	21.92	10.2%
	La Madeleine^¶^*	—	—	10.16	31.1%	15.34	14.5%
	EH^+¶^	26.15	25.4%	10.37	30.5%	17.87	12.5%
^2^Vcd	Ostuni 1b	4.62		2.44		1.17	
	Lagar Velho	79.46	5.8%	—	—	33.50	3.5%
	La Madeleine*	—	—	17.50	13.9%	22.73	5.1%
	EH	46.02	10.0%	14.72	16.6%	18.12	6.5%
^3^Vc	Ostuni 1b	28.99		12.91		8.55	
	Lagar Velho	116.52	24.9%	—	—	59.19	14.4%
	La Madeleine*	—	—	29.10	44.4%	39.74	21.5%
	EH	77.84	37.2%	27.12	47.6%	38.20	22.4%
^4^Sedj	Ostuni 1b	52.81		27.87		14.02	
	Lagar Velho	80.83	65.3%	—	—	49.41	28.4%
	La Madeleine*	—	—	31.66	88.0%	44.75	31.3%
	EH	68.05	77.6%	34.07	81.8%	42.37	33.1%

^1^Ve = Volume of the enamel cap, mm^3^; ^2^Vcd = Volume of the coronal dentine, mm^3^; ^3^Vc = total volume of the crown, mm^3^; ^4^Sedj = surface area of the enamel-dentine junction, mm^2^; ^+^Bayle *et al*.^35^; ^¶^Bayle *et al*.^34^; *estimates affected by occlusal wear^34^.

The dental tissue proportions of Os1b represent the fraction of the crown formed *in utero* before the death of the individual. The two central incisors are roughly comparable in terms of relative crown formation. The observed differences are attributable to their different morphology, as well as to some loss of enamel on the lateral and cuspal portions of the buccal aspect of the lower tooth (Fig. 2b). The lower volume attainment of LRi2 reflects the later initiation of this crown’s mineralization in respect to the mineralization times of the central incisors^36, 37^. The EDJ surface of LRi1 and ULi1 is close to 80% of the final area of the modern reference sample, thus suggesting that the two teeth were close to the end of the recruitment of new ameloblasts along the EDJ.

## Discussion

**Figure 4:**
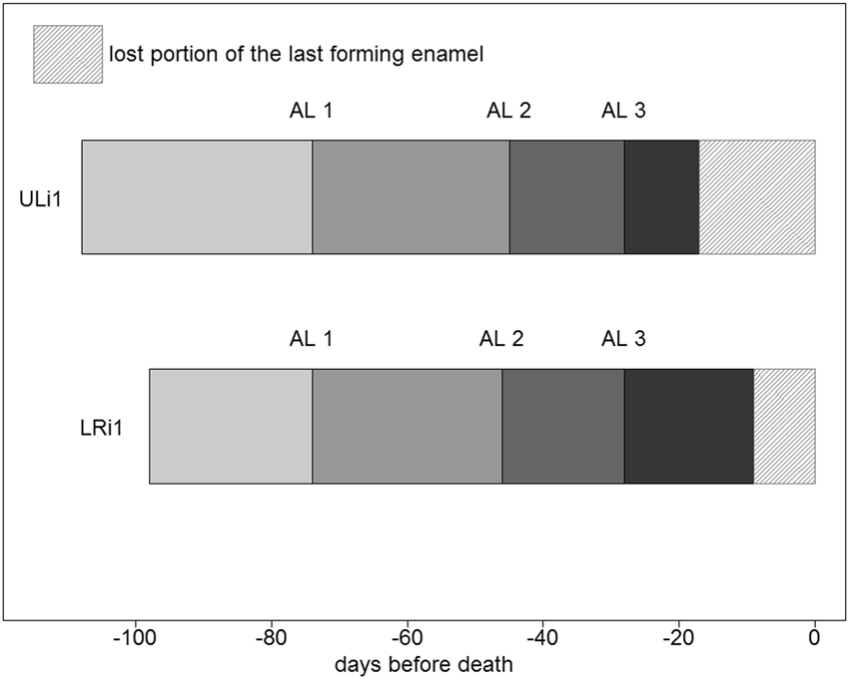
Schematic representation of the time elapsed between each of the biological landmarks (AL 1, AL 2, and AL 3) in the two central incisors of the Os1b individual. The three Accentuated Lines are aligned in order to estimate more precisely the prenatal Crown Formation Time. See text and Supplementary information online for further details.

The raw results of the CFT calculations show a difference of ca 3 days between the crowns of the two central incisors, with the upper crown taking the longest time to form (Table 1). A detailed overview of the time elapsed between each of the accentuated incremental lines (indicative of periods of systemic stress; labeled AL 1, AL 2, and AL 3 respectively) highlights a strong correspondence in time span between the three ALs in the two crowns (see Table 1 and Fig. 4). Therefore, the three ALs can be considered as fixed cross-references between the two crowns, representing the same set of stress events in the fetus’ life, and allow a reliable reconstruction of the crowns’ chronology (Table 1 and Fig. 4).

After the alignment of the ALs (Fig. 4), and after the correction of the CFTs (see Supplementary Methods) the ULi1 starts to form 10 days before the LRi1. Looking at the last formed enamel, a difference of 10 days between the two crowns is observable between AL 3 and the end of the formation of the enamel. Because the end of crown formation represents the death of the individual, this difference should be nil or zero. The most plausible explanation is that the crown of ULi1 lost an additional portion of the cervical enamel (Fig. 4) due to post-depositional processes^28, 38^. Consequently, the total CFT of the ULi1 can be adjusted to 108 days and the total CFT of the LRi1 to 98 days (Table 1, Fig. 4, Supplementary Methods online). Therefore, the most parsimonious assessment of the chronology of the three stress events, in days before death, is 28 days for AL 3, 45–46 days for AL 2, and 74 days for AL 1.

The AL that forms at birth, known as the Neonatal Line (NL)^39^, is generally used as a reference point when reconstructing dental chronologies^28^. In a fetal individual, however, the absence of this biological marker inhibits the direct assessment of the Crown Initiation Time (CIT). Birch^40^ and Birch and Dean^37^ offer a detailed review of known CITs in the deciduous dentition and conclude that the 95% confidence interval for initial mineralization for the mandibular central incisors is between 17 and 19 gestational weeks post-fertilization, assuming a mean full-term gestation length of 39 weeks (figures for the central maxillary incisors are not provided by the same authors). Therefore, in order to derive the age at death of Os1b, the CFT of the LRi1 has been added to the Birch and Dean^37^ reported CIT. We consider this latter to be the most reliable CIT estimate available in the literature based on dental enamel histology. Thus, the age at death of Os1b can be framed in the interval of 31 (17 for the time prior to mineralization + 14 weeks of CFT) and 33 (19 weeks for the time prior to mineralization + 14 weeks of CFT) gestational weeks. Based on other published crown initiation times for the mandibular central deciduous incisors (Supplementary Table S2, values from Birch and Dean^37^), the chronological age at death estimate for Os1b never exceeds the 33 weeks.

The skeletal age at death estimate for Os1b ranges between 34 and 36 gestational weeks^23^. If the skeletal age was correct, the mineralization of the LRi1 should have been initiated between 34−14 = 20 and 36−14 = 22 gestational weeks. These values are outside the known ranges reported in the literature^37^ (in which the latest crown initiation time = 19 weeks). Moreover, even if there are no comparative data, as far as we know, for the volumes of incomplete deciduous crowns, the rather small percentage of crown tissue volumes measured in Os1b (Table 2) reinforces the reliability of the present results. The observed 80% of EDJ surface already formed at death apparently contradicts these results. However, given the expected deceleration of the enamel extension rate in the cervical region^31^, the remaining 20% of EDJ surface to complete the crown would correspond to a larger proportion of the total CFT. Data collected via virtual histology suggest a 95% confidence interval of Os1b age at death between 30.7 and 34.3 gestational weeks (see Table 1 and Supplementary Table S2). Conversely, the skeletal age at death assessment by Vacca and coworkers^23^ has a 95% confidence interval of 32–38 gestational weeks (Supplementary Table S1). Even considering the potential for error, the difference between these two estimates is large from a gestational (early developmental) perspective. This discrepancy suggests that there is a substantive difference between these two estimators. This conclusion is further supported by the fact that the age at death estimates derived from Os1b’s crown heights^41, 42^ (34–38 gestational weeks; Supplementary Table S1), based on modern references, are comparable to the age at death estimates derived from modern skeletal standards.

The estimate for age at death for Os1b through virtual histology describes an individual in a preterm developmental stage^43^. These results lower the age at death of the fetus in respect to the near term gross morphological estimate by Vacca and coworkers^23^. The discrepancy between the two assessments suggests that modern fetal skeletal growth standards^24, 44^ are less accurate than the dental chronologies method adopted in this paper^28, 31^. The almost exact match of the relative chronologies for the ALs in the two central incisors of Os1b, reinforces the idea that this histological age at death estimate is accurate. Consequently, it is plausible to conclude that the difference observed between the osteological^23^ and the chronological age of Os1b, suggests a fetal developmental timing for this Upper Paleolithic individual that is slightly faster than in modern fetal individuals^24^, although pathological conditions accelerating the fetal skeletal growth^45^ or decelerating the enamel secretion cannot be excluded.

Variations between skeletal, dental and chronological age have been observed through the whole Pleistocene^46, 47^. Os1b shows a skeletal and dental development (Supplementary Table S1) that is more advanced than a modern fetus of the same gestational age, as derived from the present contribution. This evidence reinforces the hypothesis that developmental rates have varied through time^47^. Collectively, these findings support the idea that modern growth standards may be inadequate when analyzing archaeological remains.

The virtual histomorphometric analyses of the prenatal enamel of Os1b highlight the presence of three physiological stress events, which rarely occur during the sheltered life *in utero*. The chronology of these stress events reveals three severe episodes that disrupted the enamel development of the fetus during the last two and half months of life. However, the stress events did not influence the dental growth trajectories, which remained constant (Fig. 3).

The Ostuni 1 burial represents an exceptional finding, which directly speaks to the death of both a mother and a fetus during pregnancy. This is only rarely observable in the paleoanthropological record. Human childbirth is known to be difficult and risky in many cases. The so-called obstetrical dilemma is invoked as a major problem in the evolution of human childbirth. To date, a combination of anatomical, physiological, developmental^48^, evolutionary^49^, and cultural phenomena^50^ have been used to explain the multifactorial complications associated with childbirth. It is worth noting that the antero-posterior and transverse diameters of the pelvic inlet of Os1 (110 mm and 129 mm respectively^23^) are comparable with current modern and archaeological pelvic inlet measurements^50^, thus excluding the pelvic dimensions as a factor complicating the Os1 pregnancy. Moreover, intrauterine growth restriction (i.e. when the fetus does not reach its growth potential; this is a condition that is associated with perinatal morbidity and mortality and, if the Barker Hypothesis is correct, with the development of diseases later in life^51–53^) should be excluded as a possible cause of death for Os1b, as indicated by its fast skeletal growth^23^ and by its normal dental growth trajectories (Fig. 3). The presence of three enamel ALs (Fig. 2c,d and Table 1) suggest that both the Ostuni 1 mother and fetus were under severe physiological stress during the last two and half months of pregnancy. These stressors possibly resulted in the death of both the mother and the child.

This paper suggests that the use of modern reference standards^24^ in the osteological analysis of human pre-industrial fetal remains is potentially misleading and highlights the need to find new skeletal and dental standard references targeted for archaeological, and paleoanthropological, specimens. The (virtual-) histological approach, paired with osteological analyses, offers a methodology to create new population-specific standards aimed at partly overcoming the possible discrepancy between biological and chronological age at death estimates.

## Methods

### Virtual histomorphometry of dental enamel

Tooth crowns permanently record growth information, allowing for the reconstruction of precise tooth crown formation times. In addition to this, they record physiological stress events experienced during development. In infants whose deciduous teeth and/or first permanent molars are still forming, age at death can also be determined^28, 37, 54^. Dental ontogenetic studies in primates rely on the rhythmic growth of enamel, which produces short and long period incremental markings, visible at the microscopic level^55, 56^. The secretion of the enamel matrix is subject to inner biological rhythms. Consequently, dental enamel thin sections can be used to examine circadian growth markers (cross striations) along the enamel prisms, as well as long period markers (regular Retzius lines or brown striae) with circaseptan (6–12 days in humans^57, 58^) periodicity (see the extensive literature review on enamel increments given in Hillson^55^).

Severe physiological stress can produce a disruption of the matrix secretion in the corresponding position of the developing ameloblast front, corresponding to accentuated brown striae, known as Accentuated Lines^55^. The Neonatal line (NL) is generally the first AL, characterizing all the deciduous teeth and the first permanent molars of individuals that survive the perinatal stage; this birth marker separates the tissues formed prenatally from those growing after birth^39, 59^, and can be used as an indicator of birth survival and as a reference point for deciduous crown formation chronologies^28^.

The enamel developmental parameters are generally estimated destructively through histological analyses of thin sections^32, 37, 60, 61^. Recently, there has been an exponential increase in, and technological development of, high resolution (both spatial and contrast) tomographic techniques – such as phase-contrast SRμCT^8, 10, 25–27, 62^ and also μMRI^63, 64^ – for the estimate of crown and root formation times in fossil specimens.

The spatial resolution achieved for the Os1b teeth SRμCT measurement (pixel size = 7.7 μm) does not allow for the visualization of the daily cross striations, which range between 2.4–5.7 μm^31, 65, 66^. The prenatal Crown Formation Time (CFT) and the chronology of the ALs in Os1b were calculated following the method described by Guatelli-Steinberg *et al*.^32^ and by Birch and Dean^37^, where the CFT is derived from a sequence of prism lengths measured across isochronic biological landmarks. The Nava and coworkers^31^ regression formula, targeted for the prenatal enamel of the central deciduous incisors and derived from the pre-industrial skeletal series of Velia, was adopted to estimate the prenatal CFT from the prism lengths.

The prenatal CFT variation profiles with reference to the EDJ, shown in Fig. 3, were calculated with a locally weighted polynomial regression^67^ fit of the lengths on the EDJ against the prenatal CFTs (data from Table 1 and from Nava *et al*.^31^).

All data analyses and graphs were performed using the R statistical package version 3.3.2^68^.

### Synchrotron X-ray µCT data acquisition

The tooth crowns of the ULi1 and the LRi1 were imaged by means of SRμCT at the SYRMEP beamline^69^ of the Elettra - Sincrotrone Trieste laboratory in Basovizza (Trieste, Italy). Sample radiographs (1440 projections over a 180° total scan) were acquired using a water-cooled, 16 bit, 2048 × 2048 CCD camera with an effective pixel size of 3.85 μm. We employed a monochromatic X-ray beam with an energy of 30 KeV, a sample-to-detector distance of 150 mm, an exposure time/projection of 3 sec applying a binning = 2 × 2 to the detector pixels. Axial slices were reconstructed with an isotropic voxel size of 7.7 μm using the filtered backprojection algorithm through the SYRMEP Tomo Project (STP) software^70^. The final dataset used to generate the virtual histological slices (77 μm thick) was produced applying a single-distance, phase-retrieval algorithm^71^ to the sample projections setting the δ/β ratio to 40. A sharpening filter was applied to the projections before to start the reconstruction procedure.

The whole right hemimandible containing the LRi2 tooth was imaged by microfocus X-ray CT (focal spot size of the source: 5 μm) at the TomoLab station of Elettra^72^. A set of 1800 projections over a 360° total scan were acquired using a water-cooled, 12 bit, 4008 × 2672 CCD camera with an effective pixel size of 12.5 μm. The scan was carried out with a polychromatic X-ray source (Voltage: 90 kV, current: 88 μA), a source-to-sample distance of 300 mm, a source-to-detector distance of 400 mm, an exposure time/projection of 8 sec applying a binning = 2 × 2 to the detector pixels. The whole sample has been imaged in two separate scans and the reconstructed axial slices were then stitched. The slice reconstruction was done with an isotropic voxel size of 18.7 μm by the commercial software COBRA (Exxim, USA). The same software was used for beam hardening artifacts correction. The *Pore3D* software^73^ was applied to the reconstructed axial slice for ring artifacts removal.

## Electronic supplementary material

Supplementary Information
