# Supplementary material for: Virtual histological assessment of the prenatal life history and age at death of the Upper Paleolithic fetus from Ostuni (Italy)
Source: Sci Rep. 2017 Aug 25;7:9427. doi: 10.1038/s41598-017-09773-2 (PMC5572742; doi:10.1038/s41598-017-09773-2)
Supplement: Supplementary file 1 — Supplementary Information [file 41598_2017_9773_MOESM1_ESM.pdf]

Supplementary Information

Virtual histological assessment of the prenatal life history and age at death of the Upper Paleolithic fetus from Ostuni (Italy)

Alessia Nava[1,2*], Alfredo Coppa[1], Donato Coppola[3,4], Lucia Mancini[5], Diego Dreossi[5], Franco Zanini[5], Federico Bernardini[6,7], Claudio Tuniz[6,7,8], Luca Bondioli[2].

[1]Dipartimento di Biologia Ambientale, Università di Roma 'La Sapienza', Rome, Italy. [2]Servizio di Bioarcheologia, Museo delle Civiltà, Rome, Italy. [3]Università degli Studi di Bari Aldo Moro, Italy. [4]Museo di "Civiltà preclassiche della Murgia meridionale", Ostuni, Italy. [5]Elettra - Sincrotrone Trieste S.C.p.A., Basovizza (Trieste), Italy. [6]Centro Fermi, Museo Storico della Fisica e Centro di Studi e Ricerche "Enrico Fermi", Piazza del Viminale 1, 00184 Roma, Italy. [7]Multidisciplinary Laboratory, The "Abdus Salam" International Centre for Theoretical Physics, Strada Costiera 11, 34014 Trieste, Italy. [8]Centre for Archaeological Science, University of Wollongong, Northfields Ave, Wollongong, NSW 2522, Australia. *Correspondence and requests for materials should be addressed to A.N. (email: alessia.nava@uniroma1.it)

Supplementary Table S1: Summary table of Vacca *et al.'s* (2012) assessment of Os1b's developmental age. All estimates are based on Fazekas & Kósa (1978), except when specified. CI=Confidence Interval.

**Bone**	**side**	**dimension**	**value (mm)**	**Developmental age (gestational weeks)**
frontal	L	length	42.5	32
frontal	L	width	33.6	28
parietal	R	height	52.5	32
parietal	R	width	62.3	32 - 34
occipital *pars basilaris*	-	length	14.1	28 - birth
occipital *pars basilaris*	-	width	11.9	28 - birth
occipital *pars lateralis*	L	length	20.1	28 - birth
occipital *pars lateralis*	R	length	20.0	28 - birth
occipital *pars lateralis*	L	width	13.0	28 - birth
occipital *pars lateralis*	R	width	13.7	28 - birth
temporal squama	L	height	21.7	32 - 34
body of the sphenoid	-	length	10.8	32 - 40

body of the sphenoid	-	width	15.9	32 - 40
lesser wings of the sphenoid	R	length	15.7	32 - 40
lesser wings of the sphenoid	L	length	15.8	32 - 40
lesser wings of the sphenoid	R	width	9.0	32 - 40
lesser wings of the sphenoid	L	width	8.6	32 - 40
greater wings of the sphenoid	L	length	26.0	32 - 40
greater wings of the sphenoid	L	width	18.0	32 - 40
zygomatic	L	length	21.8	32 - 36
zygomatic	R	length	20.2	32
zygomatic	L	width	17.7	>36
zygomatic	R	width	17.8	>36
nasal	R	length	7.8	28
nasal	R	width	5.0	30 - 32
mandible	L	length (total)	43.2	>36
mandible	L	length (body)	33.2	>36
mandible	R	length (body)	33.4	>36
humerus	L	maximum length	58.5	36 - 38
humerus	R	maximum length	59.2	36 - 38
humerus	L	maximum length	58.5	36 - 38
humerus	L	maximum length	58.5	35.6 ±2.33*
ulna	L	maximum length	56.3	>38
ulna	L	maximum length	56.3	36.4 ±2.2*
radius	L	maximum length	49.0	>38
radius	R	maximum length	49.5	>38
radius	R	maximum length	49.5	36.5 ±2.29*
neural arch of the atlas	-	length	11.9	36
Rib IV	L	length	49.4	34
Rib IV	R	length	48.4	34
Rib VII	L	length	55.6	34 - 36
Rib VII	R	length	54.7	34 - 36
ileum	L	length	26.7	34 - 36
ileum	R	length	27.4	34 - 36
ileum	L	width	25.6	34 - 36
ileum	R	width	25.5	34 - 36
ischium	L	length	14.6	34 - 36
ischium	R	length	14.4	34 - 36
ischium	L	width	9.7	34 - 36
ischium	R	width	9.7	34 - 36
pubis	L	length	12.9	34 - 36

pubis	R	length	12.9	34 - 36
femur	L	maximum length	61.0	34 - 36
femur	L	maximum length	61.0	33.7 ±2.08*
tibia	R	maximum length	57.2	36
tibia	R	maximum length	57.2	35.5 ±2.12*
**Deciduous tooth**	**side**	**dimension**	**value (mm)**	**Developmental age (gestational weeks)**
upper central incisor	L	crown height	4.3	35[¶]
upper central incisor	R	crown height	4.2	34[¶]
upper lateral incisor	L	crown height	3.6	35[¶]
upper lateral incisor	R	crown height	3.9	37[¶]
upper central incisor	L	crown height	4.3	36 (33 - 40 95% CI)[§]
upper central incisor	R	crown height	4.2	35 (32 - 39 95% CI) [§]
upper lateral incisor	L	crown height	3.6	37 (34 - 41 95% CI) [§]
upper lateral incisor	R	crown height	3.9	38 (35 - 43 95% CI) [§]

*Value calculated with the linear regression formulae from Scheuer *et al.* (1980).

[¶] Value calculated with the Deutsch et al (1984) regression formula.

[§]Values obtained with Olivares *et al*. (2014) regression formula.

References

Deutsch, D., Tam, O. & Stack, M. Postnatal changes in size, morphology and weight of developing postnatal deciduous anterior teeth. *Growth* **49**, 207-217 (1984).

Fazekas, I. G. & Kósa, F. *Forensic fetal osteology*. (Akadémiai Kiadó, 1978).

Olivares, J. I., Aguilera, I. A., Badal, J. V., De Luca, S. & López, M. C. B. Evaluation of the maximum length of deciduous teeth for estimation of the age of infants and young children: proposal of new regression formulas. *International Journal of Legal Medicine* **128**, 345-352 (2014).

Scheuer, J. L., Musgrave, J. H. & Evans, S. P. The estimation of late fetal and perinatal age from limb bone length by linear and logarithmic regression. *Annals of Human Biology* **7**, 257-265 (1980).

Vacca, E., Formicola, V., Pesce Delfino, V. & Coppola, D. in *Il Riparo di Agnano nel Paleolitico superiore* Vol. 1 (ed D Coppola) 201–364 (Università di Roma Tor Vergata, 2012).

Supplementary Table S2: Crown initiation times from the literature for the lower central deciduous incisors (Li1). Column two reports the corresponding age at death for Os1b and, when available, the 95% confidence interval. Data from Birch and Dean (2014, Table 4), Hillson (2014, Table 11), Mahoney (2012, Table 4). Data in gestational weeks.

Reference	Crown initiation time	OS1b mean age at death
Legros and Magitot (1880)	16	30.0
Broomell and Fischelis (1913)	16	30.0
Schour and Kronfeld (1938)	18	32.0
Kronfeld and Schour (1939)	18	32.0
Schour and Massler (1940)	18	32.0
Nomata (1964)	17.7	31.7
Sunderland *et al.* (1987)	16 - 19	31.5 (30.0 - 33.0)
Mahoney (2012)*	15.3	29.3
Birch (2011)	17 - 19	32.0 (31.0 – 33.0)
**Mean**		**31.2**

*value derived in weeks from the prenatal mean crown formation time, assuming a mean full term gestation length of 39 weeks

References

Birch, W. Incremental growth of deciduous tooth enamel. PhD Thesis, University College London (2011).

Birch, W. & Dean, M. A method of calculating human deciduous crown formation times and of estimating the chronological ages of stressful events occurring during deciduous enamel formation. *Journal of Forensic and Legal Medicine* **22**, 127-144 (2014).

Broomell, I. N. & Fischelis, P. *Anatomy and Histology of the Mouth and Teeth*. (Henry Kimpton, 1913).

Hillson, S. *Tooth development in human evolution and bioarchaeology*. (Cambridge University Press, 2014).

Kronfeld, R. & Schour, I. Neonatal dental hypoplasia. *The Journal of the American Dental Association* **26**, 18-32 (1939).

Legros, C. & Magitot, E. *The origin and formulation of the dental follicle* (Jansen, McClurg and co, 1880).

Mahoney, P. Incremental enamel development in modern human deciduous anterior teeth. *American Journal of Physical Anthropology* **147**, 637-651 (2012).

Nomata, N. A chronological study on the crown formation of the human deciduous dentition. *Bull Tokyo Med Dent Univ* **11**, 55-76 (1964).

Schour, I. & Kronfeld, R. Tooth ring analysis: IV. Neonatal dental hypoplasia analysis of the teeth of an infant with injury of the brain at birth. *Arch Pathol* **26**, e90 (1938).

Schour, I. & Massler, M. Studies In Tooth Development: The Growth Pattern Of Human Teeth Part II. *The Journal of the American Dental Association* **27**, 1918-1931 (1940).

Sunderland, E. P., Smith, C. & Sunderland, R. A histological study of the chronology of initial mineralization in the human deciduous dentition. *Archives of oral biology* **32**, 167-174 (1987).

Supplementary Methods: Assessment of the CFT and of the lost the portion of the cervical enamel.

The total CFT and the time span, in days, between Als, were determined following the method of Dean (2012), Guatelli-Steinberg *et al*. (2012) and using the Nava *et al*. (2017) regression formula, targeted for the prenatal enamel of the central deciduous incisors and derived from the pre-industrial skeletal series of Velia (I-II cent. CE) (Fiammenghi 2003).

For each OS1b's central incisors, starting from the tip of the dentine horn, a single prism was followed until reaching AL1. The length of this prism portion between the EDJ and AL1 was measured in micrometers and translated into days with the regression formula. AL1 was then followed back to the EDJ. Starting from this point, a new prism was identified and measured until AL2. The same procedure was repeated for AL3. The last prism was followed until the outer enamel, allowing to estimate CFT for the most cervical portion of the crown (see Figure 2).

The intersections of the ALs with the EDJ define four segments (Ci-AL1, AL1-AL2, AL2-AL3, AL3-Ce). Enamel Extension Rate (EER, i.e. the rate of differentiation of secretory ameloblasts, or the speed at which ameloblasts into the secretory front are recruited along the EDJ [Shellis 1984]) was calculated by dividing the lengths of each of the four segments along the EDJ by the corresponding number of days derived from the prisms' length (Table 1). The EERs of both teeth decelerate towards the cervix, except in the fourth segments, where the EERs have values that are outside the ranges reported in the literature for the cervical enamel (Guatelli-Steinberg *et al.* 2012, Mahoney 2015, Nava *et al.* 2017). The only plausible explanation for this is that enamel has been lost in the last forming portion of the crown. As a consequence, the number of days derived from the enamel thickness of the fourth segment of both teeth is too low to allow a consistent estimate of the corresponding EERs. Therefore, the EER of the immediately adjacent third segment can be assumed as a good proxy. In light of this, the number of days taken to form the fourth segments of both teeth has been re-estimated as the length of the fourth EDJ segment divided by the EER of the third segment for each tooth. Results are as follows: ULi1: EDJ [AL3-Ce]=590.3 µm, EER=22.9 µm day, days=26; LRi1: EDJ [AL3-Ce]=762.7 µm, EER=27.7 µm day, days=28.

The original enamel formation estimates for the fourth segment were 11 days for the ULi1 and 19 days for the LRi1 (see Table 1). After the re-evaluation of the EERs in the fourth segments of both teeth, an additional 7 days were added to the CFT of the ULi1 and an additional 9 days were added to the CFT of the LRi1. After the alignment of the ALs, an additional 10 days were added to the ULi1 CFT, on top of the 7 already added (see text for details). Therefore the post-depositional loss of enamel secretion is quantifiable to 17 days for the ULi1 and 9 days for the LRi1 (Fig. 4). Consequently, the total CFT for the ULi1 equals 108 days and total CFT for the LRi1 equals 98 days.

References

Dean MC. A histological method that can be used to estimate the time taken to form the crown of a permanent tooth in *Forensic Microscopy for Skeletal Tissues. Methods in Molecular Biology (Methods and Protocols)* vol 915 (ed. Bell L.) 89-100 (Humana Press, Totowa, NJ, 2012).

Fiammenghi, C. La Necropoli di Elea-Velia: qualche osservazione preliminare. *Pozzuoli: Naus* (2003).

Guatelli-Steinberg, D., Floyd, B. A., Dean, M. C. & Reid, D. J. Enamel extension rate patterns in modern human teeth: two approaches designed to establish an integrated comparative context for fossil primates. *Journal of Human Evolution* **63**, 475-486 (2012).

Mahoney, P. Dental fast track: prenatal enamel growth, incisor eruption, and weaning in human infants. *American journal of physical anthropology* **156**, 407-421 (2015).

Nava, A. *et al.* New Regression Formula to Estimate the Prenatal Crown Formation Time of Human Deciduous Central Incisors Derived from a Roman Imperial Sample (Velia, Salerno, I-II cent. CE). *PLoS ONE* **12**, e0180104, doi:https://doi.org/10.1371/journal.pone.0180104 (2017).

Shellis RP. Variations in growth of the enamel crown in human teeth and a possible relationship between growth and enamel structure. *Arch Oral Biol*. **29**: 671-682 (1984).
